# Supplementary material for: Clinical Outcomes and Adverse Effects in Septic Patients with Impaired Renal Function Who Received Different Dosages of Cefoperazone–Sulbactam
Source: Antibiotics (Basel). 2022 Mar 29;11(4):460. doi: 10.3390/antibiotics11040460 (PMC9026488; doi:10.3390/antibiotics11040460)
Supplement: Supplementary file 1 [file antibiotics-11-00460-s001.zip › antibiotics-1636445-supplementary.pdf]

Supplementary Table S1. Codes for disease definitions in the study

Disease	ICD 9 code	ICD 10 code
Myocardial infarction	410-412	I20, I21, I25.2
Congestive heart failure	428	I50
Peripheral vascular disease	441, 443.9, 785.4, V43.4	I71, I79.0, I73.9, R02, Z95.8, Z95.9
Cerebrovascular disease	430-438	I60-I66, I67.0-I67.2, I67.4-I67.9, I68.1, I68.2, I68.8, I69
Dementia	290	F00, F01, F02, F05.1
Chronic pulmonary disease	490-496, 500-505	J40-J47, J60-J67
Connective tissue disease	517.1, 710, 714, 720	M05.0-M05.3, M05.8-M06.0, M06.3, M06.9, M32, M33.2, M34, M35.3
Peptic ulcer disease	521-534	K25-K28
Mild liver disease	571.2, 571.4-571.6	K70.2, K70.3, K73, K74.0, K74.2-K74.6
Diabetes without end-organ damage	250.0-250.3, 250.7	E10.1, E10.5, E11.1, E11.5, E11.9, E13.1, E13.5, E13.9, E14.1, E14.5, E14.9
Diabetes with end-organ damage	250.4-250.6	E10.2-E10.4, E11.2-E11.4, E13.2-E13.4, E14.2-E14.4
Hemiplegia	342, 344.1	G04.1, G81, G82.0-G82.2
Moderate or severe renal disease	582, 583.0-583.7, 585, 586, 588	N01, N03, N05.3-N05.6, N07.1-N07.3, N18, N19, N25
Tumor without metastasis (include leukemia and lymphoma)	140-172, 174-195, 200-208	C0-C3, C40, C41, C43, C45-C49, C5, C6, C70-C76, C80-C85, C88.3, C88.7, C88.9, C90.0, C90.1, C92, C93, C94.0-C94.3, C94.51, C94.7, C95, C96
Severe liver disease	572.2-572.4, 572.8	K72.9, K76.6, K76.6, K72.1
Metastatic solid tumor	196-198	C77-C80
Acquired immunodeficiency syndrome	042-044	B20-B24
